# Staff recognition and its importance for surgical service delivery: a qualitative study in Freetown, Sierra Leone

**DOI:** 10.1093/heapol/czaa131

**Published:** 2020-11-27

**Authors:** Chris Willott, Nick Boyd, Haja Wurie, Isaac Smalle, T B Kamara, Justine I Davies, Andrew J M Leather

**Affiliations:** 1 King’s Centre for Global Health and Health Partnerships, School of Population Health & Environmental Sciences, King’s College London, Room 2.13, Weston Education Centre, Cutcombe Road, London, SE5 9RJ, UK; 2 Bristol Royal Hospital for Children, Upper Maudlin Street, Bristol BS2 8BJ, UK; 3 College of Medicine and Allied Health Sciences, University of Sierra Leone, PMB, New England Ville, Freetown, Sierra Leone; 4 Department of Surgery, College of Medicine and Allied Health Sciences, University of Sierra Leone, 1 Percival Street, Freetown, Sierra Leone; 5 Centre for Applied Health Research, University of Birmingham, Birmingham, B15 2TT, UK; 6 Medical Research Council/Wits University Rural Public Health and Health Transitions Research Unit, Faculty of Health Sciences, School of Public Health, University of the Witwatersrand, Parktown 2193, Johannesburg, South Africa; 7 Centre for Global Surgery, Department of Global Health, Stellenbosch University, Tygerberg 7505, South Africa

**Keywords:** Sierra Leone, surgery, user fees, discretion, quality of care, staff morale

## Abstract

We examined the views of providers and users of the surgical system in Freetown, Sierra Leone on processes of care, job and service satisfaction and barriers to achieving quality and accessible care, focusing particularly on the main public tertiary hospital in Freetown and two secondary and six primary sites from which patients are referred to it. We conducted interviews with health care providers (*N* = 66), service users (*n* = 24) and people with a surgical condition who had chosen not to use the public surgical system (*N* = 13), plus two focus groups with health providers in primary care (*N* = 10 and *N* = 10). The overall purpose of the study was to understand perceptions on processes of and barriers to care from a variety of perspectives, to recommend interventions to improve access and quality of care as part of a larger study. Our research suggests that providers perceive their relationships with patients to be positive, while the majority of patients see the opposite: that many health workers are unapproachable and uncaring, particularly towards poorer patients who are unable or unwilling to pay staff extra in the form of informal payments for their care. Many health care providers note the importance of lack of recognition shown to them by their superiors and the health system in general. We suggest that this lack of recognition underlies poor morale, leading to poor care. Any intervention to improve the system should therefore consider staff–patient relations as a key element in its design and implementation, and ideally be led and supported by frontline healthcare workers.

**KEY MESSAGES**

- Patients perceive that patients who can afford to engage in informal payments to health workers, and those who have some connection to the hospital, receive higher standards of care.
- Patients are very concerned about cost and view it as the biggest obstacle to seeking and receiving high-quality surgical care.
- Staff morale is low and staff frequently feels unsupported by their seniors, by hospital leadership and by the Ministry of Health and Sanitation.
- In the context of systematic underfunding of the surgical system and low salaries, it is unsurprising that health workers engage in forms of client differentiation and the solicitation of informal payments.

## Introduction

Sierra Leone’s long civil war (1991–2002) and Ebola epidemic (2014–15) had a significant impact on its health system. Wurie et al. (2016, p. 2) describe the post-Ebola health system as ‘fragile and plagued by having inadequate [human resources for health], together with a history of low, irregular remuneration for health professionals’. Surgical services in Sierra Leone have been analysed previously (see Kingham *et al.*, 2009a,b; Kushner *et al.*, 2010, 2012; Groen *et al.*, 2012; Kwon *et al.*, 2013; Bolkan *et al.*, 2015, 2016, 2017), but to date no studies examine the experiences of providers and users of surgical services in the public system. Any attempt to improve the quality and accessibility of surgical services should be based on the perspectives of those who use the system, but for any intervention to be effective, it must also be supported by frontline providers (Walker and Gilson, 2004).

Current health indicators in Sierra Leone are among the world’s worst. Life expectancy at birth at 52.2 years is the world’s lowest (UNDP, 2018), while infant mortality is the second worst, better only than Central African Republic, and under-5 mortality is worse in only three other countries (UNDP, 2018). These poor indicators exist in the context of health expenditure that, as a percentage of GDP, was the second highest in the world in 2015 (UNDP, 2018), although from government sources alone, health expenditure is low. In 2013, for instance, only 6.8% of total health expenditure came from the Government of Sierra Leone, alongside 24.4% from donors, 7.2% from NGOs and 61.6% from out-of-pocket payments (Ministry of Health and Sanitation, 2013).^1^

Poverty is very high in Sierra Leone; 40.1% of Sierra Leoneans earned less than the World Bank’s $1.90 per day poverty line in 2018, though this is lower than a number of other sub-Saharan African countries for the latest year that figures are available [World Bank, n.d.(a)]. Income per capita at $435 is also one of the lowest in the world [World Bank, n.d.(b)].

Sierra Leoneans, particularly those living in rural areas, struggle to access surgical services of all types (Groen *et al.*, 2012; Bolkan *et al.*, 2015). In 2012, unmet need for surgery in Sierra Leone was estimated to be >90% (Bolkan *et al.*, 2015). In the same year, the country had just 0.15 surgeons per 100 000 population, lower than every country in the world for which figures are available, with the exception of Afghanistan [World Bank, n.d.(c)]. Nurse density is also one of the world’s lowest, with just 0.2 nurses and midwives per 1000 people in 2016, World Bank [n.d.(d)].

The situation in the capital, Freetown and the surrounding Western Area, is considerably better than elsewhere, but Bolkan *et al.* (2015) calculated that even in the Western Area, unmet surgical need stood at 82.1%. Aside from specific patient groups, most notably pregnant and lactating mothers and children under 5, who are covered by Sierra Leone’s free healthcare initiative (Pieterse and Lodge, 2015), all surgical care in government facilities is paid for out-of-pocket.

Our study seeks to understand views on and experiences of the surgical system in the Western Area from the perspective of both providers and users of surgical services. In part this is to shed light on processes of care: how do patients enter and make their way through the surgical system, and what barriers prevent them from doing so; and partly it is to illustrate diverse views on why the system works as it does and what effects this has on those operating within it. This paper is part of a larger piece of research, the purpose of which is to develop intervention(s) to improve access and quality of surgical care in this part of Sierra Leone.

## Materials and methods

We conducted qualitative research in one tertiary referral hospital, two secondary hospitals and six primary care sites (Peripheral Health Units, PHUs) in the Western Area of Sierra Leone, which encompasses Freetown and its environs. This paper draws on interviews (*N* = 66) carried out with health care providers in these facilities and interviews (*N* = 37) with patients or those with treatable surgical conditions, which were conducted between May 2018 and August 2019. The patient group was split into two smaller sub-groups: those who used the public system and completed care (*N* = 18) and those who began care in the public system but did not complete care (*N* = 6). We further interviewed a group of people who were suffering from a condition that was amenable to surgical care, but who had chosen not to use the public system (*N* = 13). Finally, we conducted two focus group discussions (FGDs), one each with heads of PHUs in the urban (*N* = 10) and rural (*N* = 10) districts of the Western Area, respectively. The data were collected by two of the authors of the paper and three research assistants, under the supervision of the lead author. See Table 1 for information on the category and location of interviewees.

**Table 1:** Health workers interviewed, by category, location and gender

	Tertiary hospital	Secondary site	PHU
Nurse	30 (6 male, 24 female)	5 (1 male, 4 female)	9 (5 male, 4 female)
Junior doctor	5 (3 male, 2 female)	2 (2 male, 0 female)	0
Senior doctor/Consultant	10 (9 male, 1 female)	1 (1 male, 0 female)	0
Other health worker	3 (2 male, 1 female)	1 (0 male, 1 female)	0

The tertiary centre was chosen because it is the main public hospital providing adult surgical services in Freetown and receives patients from across the country. The two secondary sites were included because they are two Freetown-based hospitals that refer surgical and other cases to the tertiary centre. The six PHUs were purposively chosen through a combination of geographical area and size of facility. PHUs in Sierra Leone are divided into three groups, in ascending order of size: Maternal and Child Health Posts (MCHPs),^2^ Community Health Posts (CHPs)^3^ and Community Health Centres (CHCs).^4^ We chose one CHC, one CHP and one MCHP each from Western Area Urban and Western Area Rural, the two districts of the Western Area.

Sampling for the study was purposive. We interviewed health workers who had experience of treating surgical patients, on both the wards and in theatre, and those who worked in facilities delivering little or no surgical care but who had experience of referring surgical patients to larger facilities. Most patients were recruited from among those who were admitted to the surgical wards at the tertiary hospital. They were approached while in the hospital and the study was introduced. These patients were contacted one month after they left the hospital and the study was explained in more detail, followed by the formal consent process. Informed consent was gathered from every participant through the use of participant information and consent forms, which participants had 24 h to examine before agreeing to participate. No patients declined to participate.

Those who had not sought care were identified by PHU staff. PHUs are firmly embedded in communities, so staff at these facilities could be expected to know people who were suffering from surgical conditions even if they had not formally sought care. FGDs and interviews with health workers took place at their workplaces or at the tertiary centre. Interviews with patients and those with untreated surgical conditions took place in their homes. In the case of those who had accessed surgical services, the interviews took place at least 30 days after they had left the hospital. The only exclusion criterion for both providers and users was people under the age of 18. There was no maximum age.

We chose the three different user groups because we wanted to understand experiences of and views on the surgical system from a variety of perspectives. We were aware that poorer patients frequently do not complete care, so were keen to ensure that we interviewed this group of people, to reduce the chances of introducing socio-economic bias into recruitment. We interviewed those who had not sought care in the public system because they could provide a unique perspective on perceived barriers to care, having felt unable to access the public system despite suffering from a condition that was amenable to surgical care.

The interviews with health workers covered a variety of issues, including their job satisfaction, perceived barriers to care-seeking in the community, their relationships with both colleagues and patients, and perceptions of how well or badly their work was recognized by patients and by their superiors. Interviews with patients covered their experiences of seeking care and their choice of facility, experiences once inside the system, relationships with care providers and perceptions on how the surgical system could improve. Interviews with those who had not sought surgical care in the public system covered their reasons for choosing not to seek this type of care and their experiences of using informal providers or self-care. They also addressed issues around perceptions of how the public system could be made more accessible to them.

Interviews and FGDs took place in both Krio and English, depending on the participants. The majority was in Krio. The interviews and focus groups were audio recorded. English interviews were transcribed verbatim and Krio interviews underwent a simultaneous translation and transcription. They were then inputted into NVivo version 12, where they were analysed using a thematic analysis approach.

Ethical approval was obtained from the ethics committees of the authors' institutes.

## Results

As noted above, interviews with providers and users of services covered a wide variety of different issues related to experiences of care in the surgical system. Three themes were particularly prominent in participants’ responses: relationships between staff and patients, costs of care and low staff morale. These themes therefore form the basis of our results.

### Staff–patient relations

Perceptions of relationships between staff and patients vary considerably, both within the patient group and between patients and providers. Most providers, particularly those working in the PHUs, characterized their relationships with patients as positive:

*[I]n any health setting, the patient is the first priority. The patient is the boss, the patient is the head. That is why we are coming here every day to see that they get the satisfaction that they desire so we have a very good relationship with the patient* (in-charge, PHU, in-depth interview, IDI).

Amongst staff in the tertiary centre, the picture was more mixed, with a number of providers acknowledging that relations were sometimes strained, but placing the burden of blame for this on patients. In general, doctors would more readily acknowledge problems in relationships with patients, while nurses were more reticent. Health workers of all types also argued that all patients, aside from clinical decision-making, were treated equally. Favouritism on the grounds of personal connections or money was viewed as unacceptable:

[*T]he same care I render to the poor person is what I render to the rich person. The same care I render somebody I know is what I render to somebody I don’t know. So the care is equal, is not selective by any way* (doctor, secondary site, IDI).

However, the frequency and vehemence of comments related to staff–patient interactions from patients suggests that perceptions of positive relationships with patients and equal care were not shared by patients, who tended to perceive the care to be of poor quality and unfair towards those who are unable or unwilling to pay extra. Nurses in particular were perceived by many patients as aggressive and rude:

*The way the countenances of doctors and nurses were, you would not have the courage to even ask those questions. I used to bear with the pain I was going through rather than asking them any question* (patient who completed care).

*[I did not have a positive experience] at all because the nurses were too harsh, they have no courtesy, even to disconnect the drip when it’s finished, they do not care. I used to disconnect the drip for myself whenever it got finished. We do everything on our own and they don’t allow patients’ relatives in the ward and they are not attentive to patients* (patient who completed care).

*Most of the nurses are so aggressive … they don’t have courtesy and respect for patients at all regardless the age. They think because they are in charge of patients they can treat you like rags* (patient who did not complete care).

Not all patients received the sort of care outlined above. Our research suggests that patients perceived that there was a hierarchy of care provided by nurses at the hospital, in which different types of patients receive better or worse care dependent on their personal connections or how they behaved in the hospital. We found there was a perception that those who received better care were those who had a relative working in the hospital or who had worked there in the past, those who were able to pay informally for their care and those who purchased medicines from nurses. These patients were said to be rewarded with treatment that was solicitous and caring:

*I was satisfied with [nurses’] interaction with me because my stepmother too is a nurse; she was used to most of them. They normally ask of help from her and in responding to their request she normally tell them to take good care of me. Some of them sometimes used to come close to me and crack jokes with me* (patient who completed care).

*The people were so fast to render service to me because they realized I was someone who was once working there. I was treated at Outpatient Department in very cordial and kindly way and I was later sent to the ward. Whilst in the ward I was also given that cordial treatment because they also realized I was once their co-worker* (patient who completed care).

*[T]hey were attending to other patients, but when they noticed I was there, they prioritised me and treated me speedily because I once worked there* (patient who completed care).

In this case, the patient notes quite freely that they were prioritized due to having previously worked at the hospital, suggesting that the importance of this kind of personal connection is understood and perceived to be relatively unremarkable. There were also instances where those with no connection were treated kindly, though these were exceptions:

[*T]here was a particular nurse … who used to assist me so greatly. She was so kind that she used to ask me how I feel, and she attends to patients whenever she was called upon. She knows her job …. She never nags at any patients. There are times when a patient is asked to buy a drug and when she see that the patient’s relatives are not around to buy the drug, she buys it and the patient’s relatives will in turn pay when they come to the hospital* (patient who completed care).

*I saw doctors who used to assist a lady who couldn’t afford all that was requested. That doctor took great care until the lady improved. She was not his relative and that made me to like that particular doctor because he has humanitarian feeling for people. The lady came from the rural area and was really stranded but with the help of that doctor and one of the staff nurses, she was better* (patient who completed care).

More often, those who lacked personal connections or money were perceived to receive lower-quality care, which interviewees described as aggressive, belittling or dismissive:

*If you do not have money they will never look after you; some patients could be at the point of death but because they do not have money, they would not be looked after or be treated* (patient who did not complete care).

*What pains me … is if you give them money they come to your aid quickly but if you don’t have, you are never considered* (patient who did not complete care).

*We used to give token to the nurses and even the doctors because that is how Sierra Leone is. You are never considered or taken care of at the hospital if you don’t give them money* (patient who completed care).

### Costs of care

Within the Sierra Leonean public health system, all surgery, apart from that covered by the FHCI, is paid for out-of-pocket. In our study the most important factor to consider for patients in deciding to seek care and where to seek it was cost.

In Sierra Leone, patients must purchase everything necessary for their stay in hospital, including drips, all medication, bed fees, forms such as for registration and discharge and simple procedures such as wound cleaning and bandage replacement. Each carries a fee that may or may not include a mark-up by the staff member and for which it is very difficult for the patient to know the official cost, as costs are not readily available to patients (Phull *et al.*, under peer review). For many patients, their journey through the hospital is complicated and they need to see a variety of different staff members. At each interaction more costs are added, making the process expensive and the overall cost impossible to calculate prior to commencing treatment:

*I forgot to tell you that I paid eighty-five thousand Leones^5^ to get my discharge card not including what I paid for the bed which was seventy one thousand Leones for the days spent and another forty thousand Leones extra which I don’t know what I paid for as I speak to you* (patient who did not complete care).

This undoubtedly skews the power relation in favour of providers, who can ask for informal payments without patients knowing the official cost of a procedure. It also produces a system in which money is perceived to be the primary factor influencing the quality of care received by patients; it is perceived that those who pay extra can expect significantly better care:

*Yes I paid [25 thousand Leones for a bed]. When I paid that money, they forcefully discharged a patient because from my observations that patient was not supposed to be discharged at that moment because his operation was from his chest to his stomach. He still needed some time on the ward but being that I have given money, they had to discharge by force* (patient who did not complete care).

*And so the focus, whenever in healthcare there is a double vision of money and care, care suffers. Because everybody wants money. And so the patients who can afford get more attention. It’s human nature* (junior doctor, tertiary site, IDI).

Those who cannot (or, less likely, will not) pay are subject to the vagaries of a system over which they have little control or influence. It produces significant stress for patients, about the care they receive and how they will raise the money to pay for it. Patients commonly enter the hospital, find out how much is needed to receive care and return to their families to try and raise the money. Relatives were by far the most common source of payments for care in our study. Frequently patients raise enough money only to discover that the costs have risen with all of the sundries they must pay for:

*So after squeezing them for all these small small things, they then have to pay for surgery and it is unaffordable* (nurse, tertiary site, IDI).

Frequently, people suffering from surgical conditions chose not to enter the public health system at all due to cost implications. This was particularly the case for patients who have conditions that are not life-threatening. In these situations, the most common response to the cost of surgical care is either to try and ignore the problem or to seek some sort of care from outside the public system, most notably through traditional medicine:

*I would have taken [my grandson] to the hospital for the operation but you know that doctors now charge from five hundred thousand or six hundred thousand Leones, some are charges close to one million. But we are trying to keep some money aside so we would be able take him to the hospital for the operation. Apart from money there is no reason why we should not take him to the hospital* (relative who did not seek care for his grandson).

*Yes the major barrier is the finance. My family is not financially strong that is the reason I am still suffering from this hernia. Had it been my family is financially strong, all this story would have come to an end because I would have gone through the surgery long since* (person who did not seek care).

These findings suggest that cost of care is the major barrier to those with surgical conditions seeking care within the public system.

The statements above must also be viewed in light of the very challenging environment in which Sierra Leonean health professionals operate. The system includes a large number of nurses who have not yet received a ‘pin code’, which allows them to be paid.^6^ These nurses were an integral part of the system but received no formal remuneration whatsoever.

### Low staff morale

Staff reported that care quality and staff morale are significantly impacted by shortages of a wide variety of medicines, equipment and materials, in addition to shortages of trained staff, across the surgical system. The unavailability of equipment and medicines contributes to feelings of disempowerment and low job satisfaction. On occasion, hospital staff are required to purchase drugs and equipment themselves to carry out routine procedures:

*Most of the time we are supplied [with materials for cleaning and bandaging wounds], but it runs out so we have to buy ourselves because the supplies are limited and these materials are very important and we expect these patients at anytime so we have to be prepared for them* (nurse, PHU, FGD).

*There is equipment, but that is for delivery, wound care kits—some are bought out of pocket. For example we have to buy sutures. This all makes some in the community not to seek care at this facility because if they come late at night with a need and it is not met, they will go elsewhere* (nurse, PHU, IDI).

*It would have been nicer if we had them abundantly, so that we would not go through much strain because when we run out of an item, we won’t have to run helter skelter for them* (nurse, tertiary site, IDI).

Lack of equipment also had an impact on health workers’ perceptions of the value that the Ministry of Health and Sanitation (MoHS) and hospital management placed on them and their work. For a large number of nurses, the lack of equipment implied a lack of recognition of the work they did:

*The management should bring the materials needed to do the job but there is not enough materials in the surgical wards. When you want a good job to be done, there should be enough materials to do the job …. That is why I say I am not valued because if I am valued, they should provide all the necessary instruments and materials necessary for the job* (nurse, tertiary site, IDI).

*We want to do the job, but we do not have materials and instruments to work with like thermometers, gloves, when the children aspirates, we don’t have suction machine and there is no emergency drugs when patients come to administer to them. Even the free health care they are talking about is most times not available, that why the job is not interesting for us. You can love your job but when there is nothing to encourage you, you will never be happy doing the job* (nurse, tertiary site, IDI).

Lack of equipment and materials is perceived by health workers to be the biggest obstacle to their ability to provide care that is accessible and high-quality. Its absence leaves health workers demoralized and unable to provide the care they would like, and has also an impact on their professional ethos. Frustration at their inability to provide high-quality care contributes to lack of motivation.

Low morale is exacerbated by perceptions of a lack of recognition of their work by superiors and by the system itself. Our research suggests that nurses do not feel valued by the management of the hospital or by the MoHS. This lack of value or recognition was most frequently conceptualized in terms of the conditions under which they work: lack of medicines and equipment, long working hours, low salaries and a lack of free healthcare for themselves and their families. Nurses saw recognition embodied in both words and deeds: receiving thanks for their work was deemed to be important, as was the quality of the environment within which they worked.

*I can say we are not valued in our work area at all. They are misusing us because if you value someone, even if they don’t have much to give to that individual, there should be a little token as a sign of appreciation but nothing of the sort is happening. We are just coming to work by the grace of God because there is no salary yet since I started working* (nurse, tertiary site, IDI).

Nurses felt that the hospital management and the MoHS should show them how much they are valued by improving their working environment, through things like equipment and medication, and also relatively simple personal incentives such as free lunch, or tea for those on night shifts. Larger deficiencies that contribute to low job satisfaction include lack of access to free healthcare for health workers, low salaries and inadequate opportunities for promotion.

## Discussion

A large proportion of patients perceive their experiences at facilities providing surgical care in the Western Area to be of poor quality, expensive and unpleasant, especially for those who are unable to pay extra to receive the attention of health workers. Cost and disrespectful treatment are the main factors contributing to patients’ perceptions of poor-quality care. These two factors are inextricably linked, as ability to pay extra to health workers is viewed as the easiest way to ensure respectful care. Conversely, staff perceive that they do a good job under trying circumstances: low pay, lack of benefits and unavailability of medicines and equipment. They suggest that client differentiation on the basis of personal connections or money takes place rarely or not at all. In this section, we try to make sense of the relationships between health workers and surgical patients in the Western Area and to understand why care can be sub-optimal.

A number of explanations have been forwarded for disrespectful or aggressive care by health workers in sub-Saharan Africa, such as it being part of a process of status positioning (Jewkes *et al.*, 1998; Andersen, 2004), to force patients to cooperate (Rominski *et al.*, 2017) or because senior management were reluctant to sanction nurses who behaved in a disrespectful way (Jewkes *et al.*, 1998). Likewise, the influence of personal connections on access to public services is common across West Africa, and has been remarked upon extensively (see Blundo *et al.*, 2006; Smith, 2006).

When seeking to make sense of the sometimes problematic relationships between providers and users of health services, care must be taken to try and view what happens ‘from the actors’ point of view’ (Olivier de Sardan, 1999, p. 25, original emphasis) and resist imposition of externally-inspired normative judgement. One way in which we can do this is to resist a blaming narrative and instead ask not ‘why do providers not care more?’, but ‘why do providers care at all?’ The nurses in this article, predominantly female and low-paid, provide care to thousands of patients, many very poor themselves, in Sierra Leone’s surgical system every year. They put up with low or non-existent salaries, steep hierarchies and a poorly-functioning system. They are witness to the suffering of others, much of which they can do little about, with no psychosocial support system in place. Simultaneously, many health workers were very clear that they do not feel valued by the system that employs them. As Street (2016) argues, it is difficult to expect health workers like this to care for the patients they treat when they perceive that the system within which they work does not value them.

*We expect health workers to build indiscriminately with strangers the affective ties of love that would usually emerge organically within relationships of kinship, co-habitation, and conviviality. If we demand compassion be felt as a professional obligation, rather than an obligation that emerges organically within our relationships, then the wider relationships in which that professional identity is maintained and nurtured are paramount. Relationships support relationships. Recognition begets care* (Street, 2016, p. 334).

It is unsurprising that health workers, in situations such as these, resort to processes of client differentiation: providing a solicitous, attentive form of care to some patients but the opposite form to others. They may perceive that recognition is essential to providing good care, and therefore only provide it to those who show this recognition, financially or through personal connections that function as a form of kinship.

It can further be argued that patients pay the price for deficiencies within the system: health workers find it difficult to affect their working environment so they take out their frustrations on patients, particularly those who cannot contribute to them getting what they feel that they deserve. Arguments about client differentiation have their origin in Lipsky’s (1980) work on street-level bureaucracy, in which he highlights the way that frontline providers such as doctors, nurses and also non-healthcare workers seek to make sense of their work by providing high-quality service to some clients and significantly worse for others. Our findings also echo those of Andersen (2004) in Ghana, in which patients were split into two groups: those ‘good’ patients who obeyed health workers’ instructions and did not cause trouble, and ‘bad’ patients (‘villagers’) who were illiterate and confused and caused health workers stress.

### Improving the quality of care delivered

We have argued that a key factor underpinning the poor quality of care experienced by patients in the public surgical system in Freetown is problematic relationships with care providers, and further argue that lack of recognition of the work of these providers contributes significantly to this problem. In this section, we argue that the provision of psychosocial support to health workers would be a relatively inexpensive mechanism through which the MoHS and the University of Sierra Leone Teaching Hospitals Complex can contribute to improving staff recognition, which could have an impact on improving care quality. An intervention in this area that has been shown to work successfully in Sierra Leone is in the form of counselling and training on stress management, self-care and client care provided to health workers. The Helping Health Workers Cope project in rural Sierra Leone was shown to have had statistically significant effects on stress levels and relationships with both co-workers and patients (Vesel et al., 2015).

It is also important to examine this process from the perspective of service users, and interventions through which they can hold care providers to account. Pieterse (2019) outlines four social accountability measures that were successfully trialled in the primary care sector in rural Sierra Leone. These were community monitoring with scorecards; non-financial rewards, focused on a competition between selected health facilities; a ‘mixed methods’ approach featuring community monitoring, awareness-raising and radio-listening components and a participatory checklist with prizes. Mechanisms such as these could help to improve relationships between providers and users of services and staff morale and recognition. Staff–patient relations and level of care may be different in urban areas, particularly the Western Area, and in tertiary care, so these interventions would need to be tailored to the setting in question. However, these represent important interventions through which both provider and user challenges can be addressed.

Despite this, however, it is important to be clear that the main factors underpinning the perceived lack of recognition in our research were material: lack of availability of medicines and materials, low salaries and opportunities for promotion. This conclusion is shared by the participants in our study and by those in Street’s (2016) work. Ultimately, the material circumstances of people’s work are a core influence over their levels of satisfaction, and intervening in this area requires significant commitment from a variety of stakeholders in the Sierra Leonean surgical system and outside.

## Study limitations

A relatively small number of interviews and FGDs cannot give a representative account of the situation for surgical patients and health workers within the health system of the Western Area. The research was also completed over a relatively short amount of time, and does not provide the sort of depth that could be gained through a more immersive, ethnographic approach. Observation, which is an integral aspect of ethnographic research, could have also shed light on the differences of opinion between providers and users about their relationships.

## Conclusion

The surgical system in the Western Area provides care that is perceived by many as expensive and low-quality. Relationships between health workers and patients are frequently poor, though patients can make use of personal or financial resources to improve the quality of their care. We argue that a key factor underpinning poor-quality care is low staff morale, and a key mechanism to improve this would be through showing health workers greater recognition for the work that they do. However, participants in our study were clear that the lack of recognition lies predominantly in the material circumstances of their work. Improving staff recognition, therefore, requires understanding and commitment to improve the situation from actors both within and outside the surgical system.

## Funding

This research was funded by the National Institute for Health Research (NIHR) Global Health Research Unit on Health System Strengthening in Sub-Saharan Africa, King’s College London (GHRU 16/136/54) using UK aid from the UK Government to support global health research. The views expressed in this publication are those of the author(s) and not necessarily those of the NIHR or the Department of Health and Social Care.

## Footnotes

1 The 2014 and 2015 figures are very high due to an influx of funds to help the health system cope with post-Ebola reconstruction. Nonetheless, even before the Ebola epidemic, health spending as a percentage of GDP was significantly higher than the African average (UNDP, 2018).

2 MCPH is the lowest level of PHU. An MCHP should ideally serve a population of 500–5000 within a 5 km radius of the facility. Staffed by MCH Aides, MCHPs are often the first facility level of contact for patients. As the name suggests, services in the MCHP focus primarily on antenatal care, safe and skilled deliveries (without complications), post-natal care and child health services [Ministry of Health and Sanitation (MoHS), 2015].

3 CHPs are usually situated in a smaller town and serve a population of 5000–10 000 or more within 8 km radius of the facility. These posts have similar functions to the MCHP with added curative functions (MoHS 2015).

4 CHCs are usually situated in the chiefdom headquarter town or in a well-populated area with a catchment population of 10 000–30 000 or more within 15 km radius of the facility. The CHC has preventive and curative functions. It offers the most complex and skilled services within the primary care level of the health system (MoHS, 2015).

5 At the time that the study took place, the exchange rate was ∼11 000 Leones to the pound and 7000 Leones to the US dollar.

6 During the period in which this research took place, nurses without a pin code were removed from the hospital. This resulted in an even worse shortage of staff than existed before.
